# Factors Significantly Contributing to a Failed Conventional Endoscopic Stone Clearance in Patients with “Difficult” Choledecholithiasis: A Single-Center Experience

**DOI:** 10.1155/2014/861689

**Published:** 2014-09-30

**Authors:** Emmanuel Christoforidis, Konstantinos Vasiliadis, Konstantinos Tsalis, Dimitrios Patridas, Konstantinos Blouhos, Manousos-Georgios Pramateftakis, Moysis Moysidis, Charalampos Lazaridis

**Affiliations:** ^1^Fourth Surgical Department, Endoscopic Unit, “George Papanikolaou” Hospital, Aristotle University, Exohi, 57010 Thessaloniki, Greece; ^2^First Department of General Surgery, Section of Hepatopancreatobiliary Surgery, “Papageorgiou” Hospital, Nea Efkarpia, 56 403 Thessaloniki, Greece; ^3^Department of General Surgery, General Hospital of Drama, End of Hippokratous Street, 66100 Drama, Greece

## Abstract

The objective of this study is to retrospectively evaluate factors significantly contributing to a failed stone extraction (SE) in patients with difficult to extract bile duct stones (BDS). *Patients and Methods.* During a 10-year period 1390 patients with BDS underwent successfully endoscopic sphincterotomy. Endoscopic SE was graded as easy; relatively easy; difficult; and failed. Difficult SE was encountered in 221 patients while failed SE was encountered in 205. A retrospective analysis of the criteria governing the difficulty of endoscopic SE following the index endoscopic intervention was performed to evaluate their significance in determining failure of complete SE among patients with difficult to extract bile duct stones. *Results.* Age ≥ 85 years, periampullary diverticula, multiple CBD stones (>4), and diameter of CBD stones (≥15 mm) were all significant contributing factors to a failed SE in univariate statistical tests. In the definitive multivariate analysis age, multiple stones and diameter of stones were found to be the significant, independent contributors. *Conclusion.* Failed conventional endoscopic stone clearance in patients with difficult to extract BDS is more likely to occur in overage patients, in patients with multiple CBD stones >4, and in patients with CBD stone(s) diameter ≥15 mm.

## 1. Introduction

Common bile duct (CBD) lithiasis is present in 7%–12% of patients with cholecystolithiasis and represents a well-established indication for endoscopic retrograde cholangiopancreatography (ERCP) with endoscopic sphincterotomy and basket or balloon stone extraction (SE) techniques [1]. Bile duct stones vary in size ranging from rather small (1-2 mm) to very large (>3 cm) [2]. The vast majority of them (85%–95%) can be removed with the use of conventional endoscopic techniques [3, 4]. Balloon dilatation following endoscopic sphincterotomy is an easy to use alternative for* difficult to extract* BDS with an acceptable safety profile [5]. However, despite the refinements in endoscopic removal of BDS, complete SE can be occasionally difficult posing an endoscopic challenge.

Extraction of BDS can be difficult for anatomic alteration and stone, duct, and patients' factors [6–9]. The size and number of BDS are major determinants of their resistance to extraction. Among other important factors, the acute distal angulation of the CBD and the shorter length of its distal CBD “arm,” the altered postsurgical anatomy, and the firmness and diameter of BDS relative to the width of the distal CBD [6–9] are included.

Failure of SE exposes the patients to a substantial risk of complications, thereby increasing morbidity and mortality [10]. In such scenario, further treatment is mandatory to avert life threatening complications [11]. Consequently, if endoscopic SE fails, patients are often referred for surgery even in the presence of substantial comorbidity [12]. The need for a definitive minimally invasive management of difficult to extract BDS forced the medical community to develop several therapeutic fragmentation modalities through shock waves both internally and externally. However, these methods are not widely available; their cost and indications are still under clinical evaluation [13]. Despite the fact that the clinical significance of factors governing the difficulty of endoscopic SE is well known, accurate identification and evaluation of these factors have attracted little investigative attention.

*The objective* of this study is to retrospectively evaluate factors significantly contributing to a failed SE in patients with difficult to extract BDS and to present the efficacy of conventional endoscopic management of difficult to extract BDS combined with biliary stenting when modern stone fragmentation modalities are not available.

## 2. Methods

### 2.1. Patients

During a 10-year period (2004 to 2014) 1390 from a total of 1448 patients with BDS underwent successful endoscopic sphincterotomy.* Among these,* 221* patients with difficult and* 205* patients with failed SE were included in this retrospective clinical study*. Patients with acute suppurative cholangitis were excluded. No oral dissolution agent was administered to the patients included in the study. Before the procedure, each patient status was classified according to the physical status classification of the American Society of Anesthesiologists (ASA) [14]. Patients with ASA score greater than 2 were excluded. Preprocedural work-up included complete blood cell count, liver function tests, serum electrolytes, and coagulation studies. All patients underwent ERCP within 24 h of admission. Patients were monitored during the procedure while sedation and analgesia were achieved with intravenous midazolam and fentanyl titrated to suit age and tolerance. General anesthesia was undertaken whenever this was necessary. An informed written consent was obtained from all patients. Diagnosis of BDS was based on radiologic and endoscopic findings.

### 2.2. Identification of Patients with Difficult Endoscopic SE-Grading of Difficulty of Endoscopic SE

Although multiple factors have been postulated to govern the difficulty of endoscopic SE, it is rather difficult to objectively determine which of them represent significant and independent contributors to the failure of endoscopic clearance. In the present study, selected literature-based criteria [5–9, 15–17], added by criteria drawn from our units experience potentially contributing to the difficulty of endoscopic SE, were used for pre- and periprocedural identification of patients with presumable difficult to extract BDS. The selected criteria are listed in Table 1.

Endoscopic retrograde cholangiopancreatography (ERCP) represents the most difficult and technically demanding endoscopic procedure. Therefore, the need to objectively evaluate the difficulty of ERCP procedures has long been acknowledged. Although several US endoscopic centers have attempted to introduce ERCP technical difficulty grading scales [18, 19], there has been no data concerning a validated, internationally acceptable, easily understandable, reproducible grading scale, which can be applied in everyday practice as a tool to objectively evaluate the difficulty of ERCP procedures and especially the difficulty of endoscopic removal of bile duct stones. Therefore, in this study we devised an easily understood difficulty grading system according to the number of attempts at CBD stone extraction with a basket or balloon catheter taking also into consideration the patient's discomfort and the feeling of difficulty by the endoscopist. Although this grading system has not been validated, it can be easily applied in everyday practice. Besides, a similar grading scale has been used in a relevant study by Kim and coworkers [6].

Following the index endoscopic session the technical difficulty of SE was graded as follows, based on number of attempts of basket grasping or balloon sweeping: easy: completely extracted by 1 to 3 attempts; relatively easy: completely extracted by 4 to 7 attempts; difficult: completely extracted by 8 or more attempts; failed: stones incompletely removed.

### 2.3. Endoscopic Procedure-Therapeutic Algorithm

All procedures of ERCP, endoscopic sphincterotomy, and SE were done by the same endoscopist with a side-viewing duodenoscope, and a standard type papillotome, using the same technique with a low osmolarity, nonionic contrast media (Iopromide, Ultravist 370: Schering, Berlin, Germany). Identified patients with a presumable difficult to extract BDS followed a specific therapeutic algorithm. The first step was the catheterization of the ampulla and injection of contrast media. In case of inability to perform a cholangiogram a guide-wire was inserted into the bile duct. Provided that the latter step was successful, an endoscopic sphincterotomy was performed, followed by attempts to extract the BDS by basket-mechanical lithotripsy and/or retrieval balloon or combination of both. All sphincterotomies were complete in type, resulting in a gush of bile and the cholangiographic confirmation of air in the biliary tree. After SE, contrast media were injected in the biliary tree and the inflated balloon catheter was withdrawn along the CBD to the duodenum to confirm clearance.* Clearance was then classified as difficult-successful*,* or failed*. In the latter event, bile duct drainage was* always* obtained by the insertion of a plastic stent (Amsterdam type stent) or a nasobiliary catheter. Stable patients with failed SE were scheduled for a repeated, elective intervention, at which time standard techniques were again implemented. Needle knife fistulotomy (NFK) was applied in cases of inability to catheterize the ampulla and was reserved only for older patients with a dilated CBD. In our unit NFK was performed by using the NK fistulotomy technique [20].

### 2.4. Collection and Evaluation of Factors Significantly Determining Failure in Endoscopic BDS Clearance

Selected preprocedural criteria added by periprocedural parameters (including parameters identified on cholangiogram and endoscopy) that contributed to the difficulty of endoscopic SE were retrospectively collected and analyzed for their significance in determining failure in stone clearance in patients with difficult to extract BDS.

### 2.5. Statistical Analysis

Statistical analysis was conducted using SPSS 20.0 (SPSS Inc., Chicago, IL). The chi-squared test for independence was used in order to study univariate relationships between failure in complete SE and binary categorical variables. Factors found to be nominally significant in exploratory univariate statistical analysis were included in the binary logistic regression model to determine the significant contributors to the failure of SE. Binary logistic regression analysis was used with a stepwise procedure for model selection in order to study covariate effects on failure in endoscopic SE in a multivariable setting. The definitive results arise from the multivariate logistic regression analysis, which estimates the independent effect of each factor after adjustment for the contributions of each of the other parameters. All tests were two-sided and *P* values less than 0.05 were considered statistically significant.

## 3. Results

All ERCPs were performed in a hospital setting. Difficult but ultimately successful SE was encountered in 221 patients while failed SE was encountered in 205 patients. The clinical characteristics and contributing factors to the difficulty of endoscopic SE in patients with difficult and failed intervention are shown in Table 2. In* all* patients with failed BDS clearance, endoprosthesis insertion establishing biliary drainage was a definitive measure. Patients with a failed index intervention underwent further endoscopic sessions that accomplished complete SE in either 2 (152/205, 74.1%) or 3 sessions (47/205, 22.9%). All patients with a failed third endoscopic session were treated successfully surgically (6/205, 2.9%).

Postprocedural ERCP-related complications occurred in 34 (7.98%) patients with difficult BDS (difficult and failed): acute cholangitis in 12; post-ERCP pancreatitis in 7; pulmonary complications in 7; acute cholecystitis in 5; and bleeding in 3. These complications were mild and were managed successfully conservatively with no ERCP-related deaths.

Following the index endoscopic intervention the selected contributing parameters were analyzed for the patients with difficult and failed endoscopic SE. Older age ≥ 85 years (*P* = 0.028), periampullary diverticula (*P* = 0.016), multiple stones >4 (*P* = 0.046), and diameter of CBD stones ≥ 15 mm (*P* = 0.012) were nominally significant contributing factors to the failure of endoscopic clearance of CBD stone(s) in exploratory univariate testing (Table 3). Presence of T-tube in situ in addition to proximal lithiasis, iatrogenic injury to the extrahepatic biliary tree in addition to CBD lithiasis, intrahepatic stones, previous gastrojejunostomy, acute distal CBD angulation, and impacted stones were not significant contributors to the failure of SE in exploratory univariate testing (Table 3). In the multivariate analysis it was found that age ≥ 85 years, odds ratio (OR) = 1.779, CI (1.110–2.850), *P* = 0.017; multiple stones >4, OR = 1.647, CI (1.060–2.559), *P* = 0.026; and CBD stone(s) diameter ≥ 15 mm, OR = 1.804, CI (1.210–2.691), *P* = 0.004, were the independent contributors to the failure of CBD stone removal (Table 4).

## 4. Discussion

Since its inception nearly 40 years ago, ERCP represents the therapeutic cornerstone for the removal of CBD stones. Following endoscopic sphincterotomy with standard extraction techniques, the vast majority of stones can be successfully removed [21]. However, in approximately 10% to 15% of patients, CBD stone removal can be difficult, predisposing to a failed endoscopic clearance [22]. Failure of SE renders the patients susceptible to the risk of cholangitis, pancreatitis, and obstructive jaundice, which add further to the significant burden of morbidity and mortality of choledocholithiasis. Therefore, assessment of contributing factors to the technical difficulty and/or failure of endoscopic SE would be extremely useful to predict a long and difficult endoscopic intervention that will require, on the one hand, special periprocedural measures, such as conscious sedation, and on the other hand, proper planning of alternative, minimally invasive therapeutic modalities, which must be carefully balanced against each other and finally with surgery, in the unfortunate event of a failed conventional endoscopic intervention. Notwithstanding, accurate determination and evaluation of factors significantly contributing to the failure of endoscopic SE have attracted little investigative attention.

Various factors have been postulated to be inversely associated with endoscopic SE, including sigmoid shaped CBD, postgastrectomy anatomy, large number of stones, intrahepatic and cystic duct stones, and stones proximal to bile duct strictures [23]. Endoscopic retrieval of bile duct stones becomes also formidable in Mirizzi syndrome and in the presence of T-tube in situ with CBD stones proximal to the T-tube [23]. However, with the exception of stone size [24], the exact role of various parameters in the success or failure of endoscopic SE remains practically unsettled.

A valuable contribution to the exceedingly sparse literature regarding the evaluation of factors contributing to the difficulty of endoscopic SE is the study by Kim and coworkers [6]. In their prospective study it was established that older age (>65 years), previous gastrojejunostomy, larger stone size (≥15 mm), impaction of stones, shorter length of the distal CBD arm (≤36 mm), and more acute distal CBD angulation (≤135 degrees) were all significant contributors to the technical difficulty for endoscopic SE. In the same study insignificant factors were found to be patients' comorbidity, previous cholecystectomy, CBD diameter ≥ 15 mm, periampullary diverticula, number of CBD stones ≥ 5, stone impaction, and stone CBD ratio > 1. Among the aforementioned significantly contributing factors, only the shorter length of the distal CBD arm and the more acute distal CBD angulation were in the definitive multivariate analysis significant and independent contributors to the difficulty of SE [6]. However, in a similar recent prospective study by García [25], no association was found between the difficulty of endoscopic SE and the shorter length of the distal CBD arm and the more acute distal CBD angulation. Furthermore, in the study by García [25], no association was found between SE and age; previous cholecystectomy and number of stones; and stone impaction and periampullary diverticula on the contrary; stone size ≥ 15 mm, CBD diameter ≥ 15 mm, and mechanical lithotripsy were all found to be significant independent contributors to the difficulty of SE.

In the present study,* which is probably the first evaluating certain factors that can significantly contribute to a failed endoscopic clearance in patients with difficult to extract bile duct stones*, following definitive multivariate analysis, it was found that age ≥ 85 years, multiple CBD stones (>4), and the diameter of CBD stones (≥15 mm) were the significant, independent contributors to a failed endoscopic BDS clearance. In addition, the present study showed that the presence of T-tube in situ and proximal lithiasis, iatrogenic injury to the extrahepatic biliary tree in addition to CBD lithiasis, intrahepatic stones, previous gastrojejunostomy, impacted stones, and acute distal CBD angulation were not significant contributors to the failure of endoscopic clearance of CBD stones.

With regard to acute distal CBD angulation, it is rather logical to hypothesize that the endoscopist will encounter technical difficulties when manipulating into an abruptly deviated common duct, just above the hepatopancreatic ampulla. This difficulty owing to the tortuosity of the distal part of CBD can cause improper positioning and inadequate manipulation of stone retrieval devices. In accordance with the study by García [25] and contrary to the study by Kim et al. [6], the present study showed that acute angulation of the distal CBD proved to be an insignificantly contributing factor to a failed endoscopic SE. In our view, acute distal CBD angulation could play a decisive role in the difficulty of endoscopic SE in the case of coexisting dilatation of the distal CBD. In such scenario, the inflated stone extraction balloon cannot sufficiently rest against the wall of the dilated duct, making the retrieval of stones extremely difficult. On the contrary, in the case of an acute angulation of a nondilated distal CBD, we believe that proper manipulations, such as the “loop” handling, following the insertion of a “Jagwire” type hydrophilic guide-wire (0.035 in, Boston Scientific) proximal to the CBD angulation, can succeed in a rather straightforward SE.

These conflicting data may be explained in part by variation in operator expertise and by the fact that ERCP is a highly operator-depended intervention. However, these intrinsic procedure-related drawbacks should not discourage investigators from further clarifying and analyzing factors contributing to a failed endoscopic SE.

The present study also showed that conventional endoscopic management of difficult to extract BDS proved for the vast majority of patients safe and effective, on the condition that bile duct drainage can be obtained and patients are capable of allowing for a repeated intervention. In fact, according to the results of the present study conventional endoscopic management cleared completely the duct in the vast majority of cases with, however, the expense of multiple treatment sessions and endoprosthesis insertion. This outcome underlines the significant role of stenting when managing patients with difficult to extract BDS, which can be lifesaving [11]. Deployment of a biliary endoprosthesis is an easy and effective alternative, especially for frail, elderly, and high risk patients. Stent insertion results in immediate biliary drainage, accomplishing treatment and/or prevention of cholangitis, serves as a “bridge” to a safe, elective, repeated session or as a dissolution therapy, and prevents impaction of stones in the distal CBD thereby maintaining the biliary flow [7, 8].

It is worth mentioning the findings of several studies which documented that after biliary stenting for a time period ranging from three to six months, a significant proportion of large CBD stones disintegrated, decreased in size, or even disappeared [26–28]. These effects of biliary stenting, which we affirm, can serve as an adjuvant measure to finally clear initially deemed “unextractable” CBD stones.

Despite the fact that in the present study conventional endoscopic management of difficult to extract BDS proved in the vast majority of patients safe and effective, this finding should not underestimate the important role of modern stone fragmentation modalities which definitively contribute to a conclusive minimally invasive treatment, especially in cases in which various conventional therapeutic strategies fail. However, one should keep in mind that the choice, if any, of which lithotripsy technique to utilize depends on availability, expertise, and certainly economic resources.

In conclusion, failed conventional endoscopic SE in patients with difficult to extract BDS is more likely to occur in overage patients, in patients with CBD stones >4, and in patients with CBD stone(s) diameter ≥ 15 mm. Endoprosthesis insertion in the event of a failed endoscopic CBD SE providing bile duct drainage offers a safety bridge to a repeated, elective, and ultimately successful minimally invasive intervention.

## Figures and Tables

**Table 1:** Selected criteria potentially contributing to the difficulty of endoscopic SE.

Anatomic variations/alterations	Stone factors	Duct factors	Patients factors
Periampullary diverticula^a^	>15 mm in diameter	Acute distal CBD angulation^b^	Age ≥ 85 years
Postgastrectomy Billroth type II or Roux-en-Y reconstruction	>4 in number (multiple stones)		
Presence of T-tube in situ + proximal lithiasis	Impacted stones		
Iatrogenic injury to the extrahepatic biliary tree + lithiasis	Intrahepatic or cystic duct stones		

^a^Defined endoscopically as a depressed lesion of 5 mm or more with intact mucosa and with the major papilla either within it or close by [17].

^b^Defined as the sharpest angle along the CBD from 1 cm below the bifurcation to 1 cm above the papilla [15].

**Table 2:** Clinical characteristics, preprocedural criteria, and periprocedural factors that contributed to the difficulty of endoscopic SE of patients with (a) difficult (*n* = 221) and (b) failed (*n* = 205) endoscopic BDS clearance.

Clinical characteristics	Failed *n* (%)	Difficult, *n* (%)
Median age in years	78,3	77,1
Gender, *n* (%)		
Men	97 (47.3)	100 (45.25)
Women	108 (52.7)	121 (54.75)
Clinical picture, *n* (%)		
Jaundice/pain	52 (25.4)	42 (19)
Abnormal blood liver test	49 (23.9)	51 (23.1)
Acute cholangitis	12 (5.8)	24 (10.6)
Acute pancreatitis	9 (4.4)	11 (64.7)
Shock	3 (1.5)	1 (0.45)
Preprocedural criteria and periprocedural factors		
Age ≥ 85	38 (38)	62 (62)
Periampullary diverticula, *n* (%)	84 (56.4)	65 (43.6)
Multiple stones > 4	136 (44.9)	167 (55,1)
Diameter of CBD stone(s) ≥ 15 mm	92 (42)	127 (58)
Presence of T-tube in situ + proximal lithiasis	25 (56.8)	19 (43.2)
Extrahepatic biliary tree iatrogenic injury + lithiasis	11 (57.9)	8 (42.1)
Intrahepatic stone(s)	8 (38.1)	13 (61.9)
Previous gastrojejunostomy	6 (35.3)	11 (64.7)
Acute distal CBD angulation	7 (46.7)	8 (53.3)
Impacted stone(s)	45 (44.6)	56 (55.4)

**Table 3:** Univariate analyses of contributing factors to failed endoscopic stone extraction.

	SE		*P* value
	Failed 205 (%)	Difficult successful 221 (%)	
Age			0.028
<85	167 (51.2)	159 (48.8)	
≥85	38 (38.0)	62 (62.0)	
Periampullary diverticula			0.016
No	121 (43.7)	156 (56.3)	
Yes	84 (56.4)	65 (43.6)	
Multiple stones			0.046
No	69 (56.1)	54 (43.9)	
Yes	136 (44.9)	167 (55.1)	
Diameter of CBD stones			0.012
<15 mm	113 (54.6)	94 (45.4)	
≥15 mm	92 (42.0)	127 (58.0)	
Presence of T-tube in situ + proximal lithiasis			NS
No	180 (47.1)	202 (52.9)	
Yes	25 (56.8)	19 (43.2)	
Iatrogenic injury to the extrahepatic biliary tree + lithiasis			NS
No	194 (47.7)	213 (52.3)	
Yes	11 (57.9)	8 (42.1)	
Intrahepatic stones			NS
No	197 (48.6)	208 (51.4)	
Yes	8 (38.1%)	13 (61.9)	
Previous gastrojejunostomy			NS
No	199 (48.7)	210 (51.3)	
Yes	6 (35.3)	11 (64.7)	
Acute distal CBD (common bile duct) angulation			NS
No	198 (48.2)	213 (51.8)	
Yes	7 (46.7)	8 (53.3)	
Impacted stone			NS
No	160 (49.2)	165 (50.8)	
Yes	45 (44.6)	56 (55.4)	

**Table 4:** Multivariate analysis of contributing factors to successful or unsuccessful stone extraction.

	OR	95% CI	*P* value
Age	1.779	1.110–2.850	0.017
Periampullary diverticula	0.684	0.452–1.034	NS
Multiple stones	1.647	1.060–2.559	0.026
Diameter of CBD stones > 15 mm	1.804	1.210–2.691	0.004

OR: odds ratio, NS: not significant.
